# Muscle Quality Assessed by the Phase Angle Can Predict Renal Prognosis in Patients with Chronic Kidney Disease

**DOI:** 10.3390/jcm14165664

**Published:** 2025-08-11

**Authors:** Yukari Mae, Tomoaki Takata, Sosuke Taniguchi, Kana Kageyama, Yudai Fujino, Shotaro Hoi, Takuji Iyama, Hajime Isomoto

**Affiliations:** Division of Gastroenterology and Nephrology, Faculty of Medicine, Tottori University, Yonago, Tottori 683-8504, Japan

**Keywords:** bioelectrical impedance analysis, chronic kidney disease, muscle quality, phase angle, sarcopenia, renal prognosis

## Abstract

**Background/Objectives**: Muscle quality reflects functional characteristics beyond muscle mass and may offer prognostic insights in patients with chronic kidney disease (CKD). This study investigated whether muscle quality predicts sarcopenia and renal outcomes in non-dialysis CKD patients. **Methods**: We prospectively recruited 93 patients with CKD (stage G1–5). Muscle quality was evaluated using phase angle (PhA) derived from bioelectrical impedance analysis. Sarcopenia was diagnosed based on criteria from the Asian Working Group for Sarcopenia. The primary renal outcome was defined as a > 30% decline in eGFR or the initiation of dialysis. Logistic regression identified factors associated with sarcopenia. Kaplan–Meier and Cox regression analyses were used to assess predictors of renal outcomes. **Results**: Lower PhA (odds ratio [OR] 0.044, *p* = 0.002) and BMI (OR 0.648, *p* = 0.015) were independently associated with sarcopenia. Sex-specific PhA cutoffs of 4.50° for men and 4.00° for women yielded high diagnostic accuracy for sarcopenia (AUC: 0.878 and 0.969, respectively). Over a median observation period of 574 days, patients with lower PhA values showed a significantly higher risk of poor renal outcomes, independent of confounding factors (hazard ratio [HR] 4.540, *p* = 0.011), even after adjustment for age, sex, BMI, systolic blood pressure, diastolic blood pressure, eGFR, and albumin. However, muscle mass was not significantly associated with renal prognosis. **Conclusions**: PhA is a non-invasive marker that may reflect muscle quality and is associated with sarcopenia and renal outcomes in CKD. It may help inform risk stratification in clinical practice.

## 1. Introduction

The global burden of chronic kidney disease (CKD) is escalating, particularly due to its high prevalence among aging populations. This trend poses a growing challenge to healthcare systems worldwide [1]. Patients with CKD often have various comorbidities, such as cardiovascular diseases, mineral bone disorders, and sarcopenia. The characteristic pathophysiology of CKD, such as uremic toxin accumulation, malnutrition, metabolic abnormality, and inflammation, can negatively affect skeletal muscle by altering its composition and function [2,3,4], leading to sarcopenia. Assessment of sarcopenia in clinical practice is necessary because it is strongly associated with quality of life and can influence the evaluation of renal function [5]. Furthermore, sarcopenia can be a risk factor for further decline in renal function and is associated with increased mortality in patients with CKD [6,7,8,9,10]. The close interplay between CKD and sarcopenia in a vicious cycle underscores the importance of early and accurate assessment of sarcopenia in patients with CKD.

Traditionally, sarcopenia is diagnosed based on muscle mass and strength [11]. However, recent consensus highlights the importance of muscle quality, which reflects the structural and functional integrity of muscle beyond quantity alone [12,13]. Muscle quality is thought to deteriorate earlier than muscle mass and may thus serve as a more sensitive indicator of early-stage sarcopenia [12,13]. Despite its clinical relevance, evaluating muscle quality remains challenging due to the need for imaging modalities such as magnetic resonance imaging (MRI) or dual-energy X-ray absorptiometry (DXA), which are often impractical in routine clinical settings. Bioelectrical impedance analysis (BIA) serves as a practical and non-intrusive tool for analyzing body composition. One key parameter derived from BIA is the phase angle (PhA), which is calculated as the arctangent of the ratio of reactance (Xc) to resistance (R). When an alternating current flows through the human body, intact cell membranes act as capacitors, delaying the current and causing a phase shift between the voltage and current. This time lag, resulting from the capacitive properties of cell membranes and tissue interfaces, is expressed as the PhA. A higher PhA generally indicates better cellular health and function, whereas a lower PhA suggests impaired membrane integrity or reduced cell mass. Specifically, a decrease in PhA reflects a reduction in Xc—primarily due to compromised cell membrane integrity or a decline in myocyte number—and is often accompanied by an increase in R caused by fatty infiltration of muscle tissue. This combination of decreased Xc and increased R indicates a deterioration in muscle quality [14,15,16]. Recent studies have demonstrated a positive correlation between muscle quality, defined as grip strength (HGS) relative to arm muscle mass, and PhA, supporting its use as a surrogate marker of muscle quality [16]. Prior studies have demonstrated associations between PhA and muscle function, nutritional status, inflammation, and mortality, particularly in patients on maintenance hemodialysis [17,18,19,20,21]. However, the clinical usefulness of PhA in evaluating sarcopenia and predicting renal prognosis in patients with CKD who are not undergoing dialysis has not been fully explored.

The objectives of this study were to (1) investigate the association between muscle quality and sarcopenia in individuals with CKD, (2) identify sex-specific PhA thresholds for sarcopenia detection, and (3) assess the predictive value of muscle quality for renal outcomes.

## 2. Materials and Methods

### 2.1. Study Population and Design

This single-center prospective study was conducted at the Department of Nephrology, Tottori University Hospital (Yonago, Japan). Patients were prospectively enrolled between April 2021 and October 2022, and each patient was followed up for renal outcomes until March 2025 or the occurrence of a renal event, whichever came first. The inclusion criteria were as follows: diagnosis of CKD and provision of written informed consent. CKD was defined as an estimated glomerular filtration rate (eGFR) < 60 mL/min/1.73 m^2^ [22] or a urine protein-to-creatinine ratio (UPCR) > 0.15 g/gCr for a period exceeding 3 months. Therefore, this definition encompasses CKD stages G1 to G5, incorporating both early-stage patients with proteinuria and those with reduced eGFR. Among 131 patients initially enrolled, 38 who met the exclusion criteria—age < 20 years, current dialysis treatment, insufficient data for sarcopenia diagnosis, or presence of implanted medical devices—were excluded, resulting in 93 patients for final analysis. Patients were followed up until March 2025, and their renal outcome, defined as a > 30% reduction in the eGFR or initiation of dialysis, was recorded [23,24]. All participants provided written informed consent prior to enrollment. The study protocol adhered to the principles of the Declaration of Helsinki and was reviewed and approved by the Ethics Committee of Tottori University Hospital (Approval No. 20A186).

### 2.2. Body Composition Analysis and Sarcopenia Diagnosis

Body composition measurements were conducted 30–60 min before non-fasting blood collection. Height was measured using a height meter (Seca 228; Seka Nihon, Chiba, Japan). Body mass index (BMI) was calculated by dividing the body weight by the square of height. Muscle mass was evaluated by assessing the appendicular skeletal muscle mass (ASM) and skeletal muscle mass index (SMI) was calculated by dividing the ASM by the square of the height (kg/m^2^). Muscle quality was assessed by PhA using MC-780A-N (TANITA, Tokyo, Japan). PhA was automatically calculated by the device from the resistance (R) and reactance (Xc) values obtained at 50 kHz using the following formula: PhA (°) = arctangent (Xc/R) × (180/π). Handgrip strength (HGS) was measured using a digital grip strength meter (GRIP-D; TAKEI, Niigata, Japan), and the maximum value among two repetitive measurements was used. Blood pressure was measured in the seated position after at least 5 min of rest using an automated sphygmomanometer. Blood pressure measurements were successfully obtained for all patients, with no missing data. Two consecutive readings were obtained, and the average values were recorded as systolic and diastolic blood pressure, respectively. Sarcopenia was assessed using HGS and skeletal muscle mass index (SMI), based on the recommendations of the European Working Group on Sarcopenia in Older People (EWGSOP) [11] and the Asian Working Group for Sarcopenia (AWGS) [25]. Diagnostic cutoff values for HGS and SMI were adopted from the AWGS criteria, with sarcopenia defined as HGS <26 kg for men and <18 kg for women, and SMI <7.0 kg/m^2^ for men and <5.7 kg/m^2^ for women. Blood and intermediate urine samples were collected after the body composition measurements.

### 2.3. Statistical Analysis

Continuous data were summarized as either mean ± standard deviation or median with range, depending on their distribution. Normality was assessed using the Kolmogorov–Smirnov test. Comparisons of clinical characteristics were conducted between patients with and without sarcopenia; intergroup differences were analyzed using χ^2^, Student’s t, or Mann–Whitney U tests, depending on their distribution. Because some variables were not normally distributed, the correlations between PhA and clinical parameters—age, BMI, HGS, ASM, eGFR, Alb, and UPCR—were analyzed using Spearman’s rank correlation coefficient. Logistic regression analysis was performed to identify the factors associated with sarcopenia. Receiver operating characteristic (ROC) curve analysis was conducted to determine the PhA cutoff value for detecting sarcopenia, and the optimal cutoff was identified using the Youden index. Kaplan–Meier survival analysis and the Log-rank test were used to compare renal outcomes between the two groups. Additionally, Cox proportional hazards regression analyses were conducted to assess factors contributing to renal outcomes. To account for nutritional status, serum albumin was included as a covariate in the Cox regression model. In both univariate and multivariate Cox regression analyses, systolic and diastolic blood pressures were also included as covariates, as blood pressure is a clinically important factor influencing renal prognosis. Variables included in the multivariate model were selected based on clinical relevance and statistical significance in univariate analysis (*p* < 0.10), except for HGS, which was excluded due to its close correlation with PhA to avoid multicollinearity. Statistical analyses were performed using StatFlex (version 6.0 for Windows, Artec, Osaka, Japan). Statistical significance was set at *p* < 0.05.

## 3. Results

### 3.1. Patient Characteristics

Of the 131 patients initially enrolled, 38 did not meet the inclusion criteria or were otherwise excluded, resulting in 93 patients being analyzed. The cohort comprised 59 male and 34 female participants, with a median age of 67.0 years (range: 26.0–89.0), as shown in Table 1. The eGFR was 42.7 (4.8–114.3) mL/min/1.73 m^2^. Body mass index (BMI) was 23.9 ± 4.1 kg/m^2^, systolic blood pressure (sBP) was 135.0 (103.0–185.0) mmHg, diastolic blood pressure (dBP) was 81.0 (45.0–102.0) mmHg, HGS was 26.9 (8.1–58.5) kg, SMI was 7.39 (5.13–10.88) kg/m^2^, PhA was 5.13 ± 0.9°, Alb was 3.90 (2.3–4.8) g/dL, and UPCR was 0.65 (0.01–11.53). Eleven patients (six men and five women) had sarcopenia. The sarcopenia group consisted of older individuals (81.5 [29.0–89.0] years), compared with the non-sarcopenia group (66.0 [26.0–84.0] years, *p* = 0.002). Additionally, the sarcopenia group showed significantly lower height (1.53 ± 0.09 m vs. 1.63 ± 0.09 m, *p* < 0.001), body weight (51.7 [34.1–58.1] kg vs. 62.9 [43.7–102.9] kg, *p* < 0.001), BMI (20.5 ± 3.6 kg/m^2^ vs. 24.3 ± 4.0 kg/m^2^, *p* = 0.005), HGS (14.0 [8.1–26.4] kg vs. 28.3 [11.1–58.5] kg, *p* < 0.001), ASM (13.7 ± 2.6 kg vs. 21.1 ± 4.5 kg, *p* < 0.001), SMI (5.56 [5.13–6.49] kg/m^2^ vs. 7.73 [6.04–10.88] kg/m^2^, *p* < 0.001), and PhA (3.87 ± 0.6° vs. 5.28 ± 0.9°, *p* < 0.001), compared with the non-sarcopenia group.

### 3.2. Correlation Between PhA and Clinical Variables

A correlation analysis using Spearman’s rank correlation coefficient revealed that PhA was significantly associated with multiple clinical parameters. PhA demonstrated a strong negative correlation with age (r = −0.615, *p* < 0.001) and strong positive correlations with HGS (r = 0.629, *p* < 0.001) and ASM (r = 0.555, *p* < 0.001). Moderate positive correlations were also observed between PhA and eGFR (r = 0.388, *p* < 0.001) and Alb (r = 0.338, *p* < 0.001). The correlation between PhA and BMI was weaker and did not reach statistical significance (r = 0.192, *p* = 0.065). No significant correlation was observed between PhA and UPCR (r = −0.254, *p* = 0.114). These findings support the association of PhA with muscle strength, nutritional status, and renal function.

### 3.3. Factors Associated with Sarcopenia

In univariate logistic regression analysis, sarcopenia was coded as 1 and non-sarcopenia as 0 in the dependent variable. Age (odds ratio [OR]: 1.070, 95% confidence interval [CI]: 1.007–1.137, *p* = 0.029), BMI (OR: 0.736, 95% CI: 0.586–0.924, *p* = 0. 008), HGS (OR: 0.809, 95% CI: 0.713–0.918, *p* < 0.001), ASM (OR: 0.550, 95% CI: 0.389–0.777, *p* < 0.001), and PhA (OR: 0.120, 95% CI: 0.038–0.379, *p* < 0.001) were associated with sarcopenia (Table 2). We further conducted multivariate logistic regression analysis to investigate the factors contributing to sarcopenia. Given that muscle strength and mass are included in the diagnostic criteria for sarcopenia and are strongly associated with each other, parameters reflecting them, such as HGS and ASM, were excluded from the explanatory variables to avoid multicollinearity. We performed binary logistic regression analysis, including age, sex, BMI, and PhA as explanatory variables, showing that BMI (OR: 0.648, 95% CI: 0.457–0.918, *p* = 0.015) and the PhA (OR: 0.044, 95% CI: 0.006–0.316, *p* = 0.002) were independently associated with sarcopenia (Table 2).

### 3.4. Diagnostic Performance of the PhA for Sarcopenia

**Figure 1:**
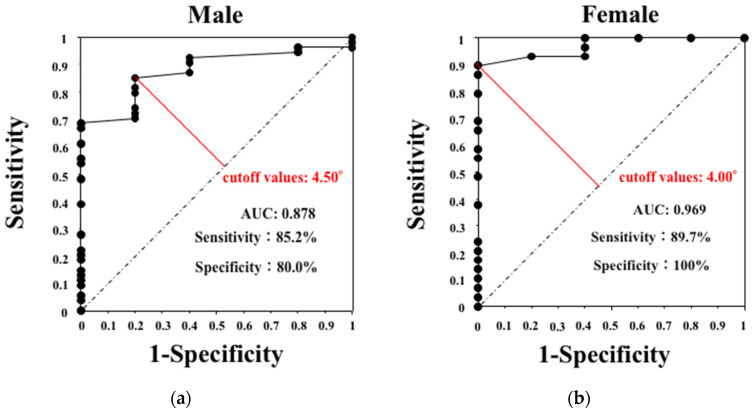
The ROC curve of PhA to detect sarcopenia. (**a**) The ROC curve for men showing an AUC of 0.878 with a cutoff value of 4.50° (sensitivity: 85.2%, specificity: 80.0%). (**b**) The ROC curve for women showing an AUC of 0.969 with a cutoff value of 4.00° (sensitivity: 89.7%, specificity: 100%). The optimal cutoff values were determined using Youden’s index. AUC: area under the curve, ROC: receiver operating characteristic, PhA: phase angle.

ROC curve analysis revealed that the PhA could detect sarcopenia, with an area under the curve (AUC) of 0.878 for men and 0.969 for women (Figure 1). Using Youden’s index, we identified the optimal PhA thresholds as 4.50° in men and 4.00° in women. These values corresponded to sensitivity/specificity rates of 85.2%/80.0% and 89.7%/100.0%, respectively. A statistical comparison of AUCs between sexes was not performed in this study. These findings indicate that the PhA has a high diagnostic ability for sarcopenia.

### 3.5. Impact of the PhA on Renal Prognosis

**Figure 2:**
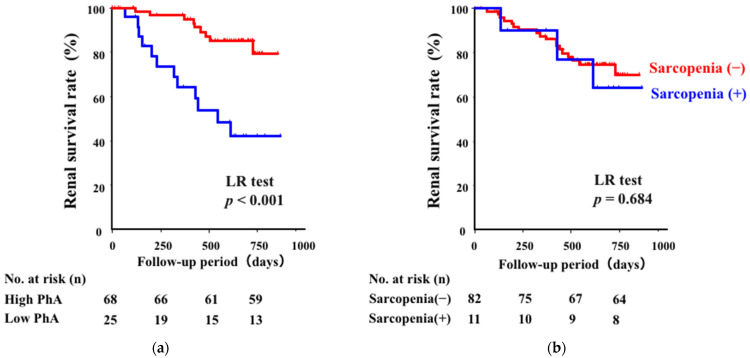
The Kaplan–Meier survival curves for renal outcomes. (**a**) The patients were divided based on their PhA. The renal survival was significantly lower in patients with low PhA (Log-rank test, *p* < 0.001). (**b**) The patients were divided based on the presence of sarcopenia. No significant difference in renal survival was observed between the groups (Log-rank test, *p* = 0.684). PhA; phase angle, LR test; Log-rank test.

Next, we investigated whether low PhA and the presence of sarcopenia could predict renal outcomes in patients with CKD. During a median follow-up period of 574 days, 21 patients experienced a > 30% decline in eGFR or initiated dialysis. To assess the predictive value of PhA, the patients were divided into “low” and “high” PhA groups based on the sex-specific cutoff values established from the ROC curve analysis (4.50° for men and 4.00° for women). Kaplan–Meier curve analysis revealed that patients with low PhA demonstrated a significantly poor prognosis, compared with those with high PhA (*p* < 0.001) (Figure 2a). During the follow-up period, renal events occurred in 12 patients (48.0%) in the low PhA group and in 9 patients (13.2%) in the high PhA group, further supporting the prognostic significance of PhA. In contrast, when the patients were divided based on the presence of sarcopenia, renal prognosis did not differ between the groups (Figure 2b).

We further conducted Cox regression analysis to assess the risk factors for renal outcomes. In the univariate model using PhA as a continuous variable, age (hazard ratio [HR]: 1.039, 95% CI: 1.004–1.075, *p* = 0.029), eGFR (HR: 0.923, 95% CI: 0.891–0.957, *p* < 0.001), Alb (HR: 0.215, 95% CI: 0.091–0.508, *p* < 0.001), and PhA (HR: 0.395, 95% CI: 0.235–0.665, *p* < 0.001) were significantly associated with renal outcomes. Neither sBP (HR: 1.015, 95% CI: 0.986–1.046, *p* = 0.315) nor dBP (HR: 0.966, 95% CI: 0.931–1.003, *p* = 0.069) was significantly associated with renal outcomes. In the multivariate model, adjusted for age, sex, BMI, sBP, dBP, eGFR, and Alb, PhA remained an independent predictor of renal outcomes (HR: 0.400, 95% CI: 0.190–0.843, *p* = 0.016), while Alb lost statistical significance (HR: 0.448, 95% CI: 0.150–1.335, *p* = 0.150) (Table 3). In addition, we performed a separate analysis using PhA as a dichotomous variable, based on the sex-specific cutoff values identified through ROC analysis (4.50° for men and 4.00° women). Patients with low PhA showed a significantly higher risk of renal events compared with those with high PhA (HR: 4.540, 95% CI: 1.417–14.54, *p* = 0.011) (Table 4). This analysis was conducted to evaluate the prognostic utility of the established cutoff values.

## 4. Discussion

This study demonstrated that PhA was significantly associated with both sarcopenia and renal prognosis in patients with CKD. We established the optimal PhA cutoff values for identifying sarcopenia in both sexes. Furthermore, we revealed that patients with low PhA were at a higher risk of poor renal prognosis, compared with those with high PhA. These findings highlight the clinical significance of PhA in the management of patients with CKD.

PhA reflects cell integrity [26,27], muscle condition [28,29], nutritional status [30,31], and inflammation [32]. As PhA reflects alterations in muscle components, it may be an early indicator of sarcopenia. This study revealed that low BMI and PhA were associated with sarcopenia in non-dialysis patients with CKD. Notably, PhA is strongly associated with sarcopenia. This study demonstrated that the OR of the PhA for sarcopenia was 0.044, indicating every 1° decrease in PhA increased the risk of sarcopenia by 22.7 times. This increase is remarkable when compared with the previous reports in patients on maintenance hemodialysis, in which every 1° decrease in PhA was associated with a 1.96 to 4.17 times increase in the risk of sarcopenia [17,18,20,21]. Given that muscle mass and function tend to be preserved in patients not on maintenance dialysis compared with those undergoing dialysis [7,33,34,35], PhA may serve as an early indicator of potential sarcopenia. We also observed that BMI was associated with the higher risk for sarcopenia. Previous studies in patients undergoing hemodialysis have shown that a 1 kg/m^2^ increase in BMI reduces the risk of sarcopenia by 26–34% [18,36] which is in line with the OR of BMI for sarcopenia observed in the present study.

In this study, PhA accurately detected sarcopenia in both sexes. We determined the PhA cutoff values for diagnosing sarcopenia: 4.50° for men and 4.00° for women. In an observational study conducted in an Asian population, the PhA was 6.55 ± 1.10° in healthy individuals. Previous studies have reported PhAs of 5.95° for young men, 5.04° for older men, 5.02° for young women, and 4.20° for older women [16]. These observations indicate that the PhA reference values may differ according to sex. Moreover, in patients on maintenance hemodialysis, PhAs of 6.20° [17] and 4.67° [18] in men and 5.70° [17] and 4.60° [18] in women predicted sarcopenia; thus, the PhA cutoff values for diagnosing sarcopenia may differ according to the patients’ comorbidities.

Importantly, our study demonstrated that PhA is a significant predictor of renal prognosis in patients with CKD. In univariate Cox regression analysis, PhA showed the strongest association with long-term renal outcomes compared to HGS and ASM, which exhibited only weak correlations. We further adjusted for potential confounders including age, sex, BMI, eGFR, serum albumin, and both systolic and diastolic blood pressure in the multivariate Cox regression model. Notably, neither systolic nor diastolic blood pressure was significantly associated with renal outcomes. Among all variables, only PhA remained independently associated with renal outcomes (HR: 4.540, 95% CI: 1.417–14.54, *p* = 0.011), whereas serum albumin lost statistical significance (HR: 0.557, 95% CI: 0.190–1.636, *p* = 0.287). These findings raise the possibility that PhA could provide complementary prognostic information to traditional nutritional and cardiovascular markers, potentially reflecting aspects of muscle cell membrane integrity and muscle quality. Kaplan–Meier analysis also demonstrated that patients with low PhA showed significantly poorer renal outcomes during follow-up, whereas no significant difference in long-term renal outcomes was observed between patients with or without sarcopenia. This lack of association may be partly attributed to the clinical characteristics of the study population. Notably, none of the patients classified as having sarcopenia had diabetic nephropathy (DN), whereas the subgroup with low PhA but without sarcopenia included a higher proportion of patients with DN. Since DN is a leading cause of CKD progression and may be associated with declines in muscle quality even in the absence of marked reductions in muscle mass or strength, PhA may more sensitively detect subtle muscle alterations linked to renal prognosis in these patients. Based on these findings that PhA potentially serves as an accurate and early indicator of renal survival in patients with CKD, we propose to incorporate muscle quality in addition to muscle mass in the assessment of sarcopenia.

Although this study suggests the relevance of muscle quality in diagnosing sarcopenia and predicting renal outcomes in patients with CKD, it has some limitations. First, it was conducted at a single center with a relatively modest sample size (*n* = 93), which may limit the generalizability of the findings. Moreover, this study analyzed the association between PhA and renal outcomes based on a single baseline measurement; longitudinal changes in PhA during the follow-up period were not assessed. As PhA may fluctuate with changes in muscle quality and overall health status, serial measurements could provide deeper insights into the dynamic relationship between muscle condition and renal prognosis. Future studies incorporating repeated PhA assessments are warranted to clarify these associations and to complement the limitations related to sample size and study design. In addition, although the primary causes of CKD differed between the sarcopenia and non-sarcopenia groups, urinary protein levels did not significantly differ. The degree of renal pathological damage was not available for all patients and thus was not included in the analysis. These factors are important determinants of CKD progression and should be incorporated in future studies to better elucidate the impact of sarcopenia on renal outcomes. Additionally, treatment strategies to prevent CKD progression may vary across institutions and individual patients, potentially influencing renal outcomes. Furthermore, since estimated glomerular filtration rate (eGFR) is calculated from serum creatinine, which is influenced by muscle mass, the presence of sarcopenia may lead to an overestimation of renal function. Although we used established equations and accounted for confounders, this potential bias should be acknowledged when interpreting the results. However, despite the limited number of subjects, associations between PhA and both sarcopenia and renal prognosis were robust, with large effect sizes and relatively narrow confidence intervals. Therefore, our observation is considered clinically relevant. Nevertheless, further multicenter studies with larger, more diverse populations are warranted to validate and expand upon these results.

## 5. Conclusions

In conclusion, this study suggests that PhA may be a clinically relevant index to assess muscle quality and is associated with both sarcopenia and renal outcomes in patients with CKD. PhA appears to provide prognostic information beyond muscle mass and strength. While these findings indicate a potential role for PhA in risk stratification, further large-scale and multicenter studies are needed to validate its utility in routine clinical practice.

## Figures and Tables

**Table 1:** Characteristics of the participants in the study groups.

Parameter	Non-Sarcopenia Group (*n* = 82)	Sarcopenia Group (*n* = 11)	*p* Value
Age (years)	66.0 (26.0–84.0)	81.5 (29.0–89.0)	0.002
Sex (male/female)	53/29	6/5	
Cause of renal disease			
Glomerulonephritis	43.4%	54.5%	
Diabetic nephropathy	18.1%	0%	
Nephrosclerosis	6.0%	27.3%	
Tubulointerstitial nephritis	6.0%	0%	
Polycystic kidney disease	3.6%	0%	
Other/unknown	22.9%	18.2%	
Height (m)	1.63 ± 0.09	1.53 ± 0.09	<0.001
Body weight (kg)	62.9 (43.7–102.9)	51.7 (34.1–58.1)	<0.001
BMI (kg/m^2^)	24.3 ± 4.0	20.5 ± 3.6	0.005
sBP (mmHg)	134.5 (103.0–102.0)	142.0 (110.0–173.0)	0.111
dBP (mmHg)	81 (45.0–185.0)	76 (68.0–91.0)	0.730
HGS (kg)	28.3 (11.1–58.5)	14.0 (8.1–26.4)	<0.001
ASM (kg)	21.1 ± 4.5	13.7 ± 2.6	<0.001
SMI (kg/m^2^)	7.73 (6.04–10.88)	5.56 (5.13–6.49)	<0.001
PhA (°)	5.28 ± 0.9	3.87 ± 0.6	<0.001
eGFR (mL/min/1.73 m^2^)	42.7 (4.8–114.3)	45.8 (21.3–85.9)	0.916
Alb (g/dL)	3.90 (2.3–4.8)	3.70 (3.3–4.5)	0.142
UPCR (g/gCr)	0.67 (0.01–11.53)	0.45 (0.04–2.81)	0.382
CKD stage			
G1–2	25.6%	27.3%	
G3a	18.3%	27.3%	
G3b	26.8%	18.2%	
G4	20.7%	27.3%	
G5	8.5%	0%	

BMI; body mass index, sBP; systolic blood pressure, dBP; diastolic blood pressure, HGS; handgrip strength, ASM; appendicular skeletal muscle, SMI; skeletal muscle, PhA; phase angle, eGFR; estimated glomerular filtration rate, Alb; albumin, UPCR; urine protein-to-creatinine ratio, CKD; chronic kidney disease. Values are presented as mean ± standard deviation (standard deviation), median (range), and percentage. Data are presented as mean ± standard deviation (SD) for normally distributed variables, median (range) for non-normally distributed variables, and percentages for categorical variables. Intergroup comparisons between sarcopenia and non-sarcopenia groups were performed using Student’s *t*-test or Mann–Whitney U test for continuous variables, depending on their distribution, and χ^2^ test for categorical variables.

**Table 2:** Factors associated with sarcopenia.

Parameter	Univariate		Multivariate	
	Odds Ratio (95% CI)	*p* Value	Odds Ratio (95% CI)	*p* Value
Age	1.070 (1.007–1.137)	0.029	0.979 (0.910–1.052)	0.558
Sex (Ref. M)	1.523 (0.428–5.424)	0.516	0.267 (0.039–1.822)	0.178
BMI	0.736 (0.586–0.924)	0.008	0.648 (0.457–0.918)	0.015
eGFR	1.003 (0.978–1.030)	0.799		
HGS	0.809 (0.713–0.918)	<0.001		
ASM	0.550 (0.389–0.777)	<0.001		
PhA	0.120 (0.038–0.379)	<0.001	0.044 (0.006–0.316)	0.002

CI; confidence interval, Ref; reference, M; male, BMI; body mass index, eGFR; estimated glomerular filtration rate, HGS; handgrip strength, ASM; appendicular skeletal muscle, PhA; phase angle. Univariate and multivariate logistic regression analyses were performed to identify factors associated with sarcopenia. Odds ratios and 95% CI were calculated. Variables included in the multivariate model were selected based on clinical relevance and absence of multicollinearity.

**Table 3:** Cox hazard ratios for renal outcomes.

Parameter	Univariate		Multivariate	
	HR (95% CI)	*p* Value	HR (95% CI)	*p* Value
Age	1.039 (1.004–1.075)	0.029	0.987 (0.944–1.032)	0.563
Sex (Ref. M)	0.838 (0.347–2.025)	0.695	0.413 (0.142–1.199)	0.104
BMI	0.999 (0.901–1.108)	0.987	0.977 (0.873–1.093)	0.637
sBP	1.015 (0.986–1.046)	0.315	0.982 (0.945–1.022)	0.376
dBP	0.966 (0.931–1.003)	0.069	1.002 (0.959–1.047)	0.929
eGFR	0.923 (0.891–0.957)	<0.001	0.929 (0.893–0.966)	<0.001
Alb	0.215 (0.091–0.508)	<0.001	0.448 (0.150–1.335)	0.150
HGS	0.955 (0.911–1.001)	0.057		
ASM	1.010 (0.918–1.111)	0.838		
PhA	0.395 (0.235–0.665)	<0.001	0.400 (0.190–0.843)	0.016

HR; hazard ratio, CI; confidence interval, Ref; reference, M; male, BMI; body mass index, sBP; systolic blood pressure, dBP; diastolic blood pressure, eGFR; estimated glomerular filtration rate, Alb; albumin, HGS; handgrip strength, ASM; appendicular skeletal muscle, PhA; phase angle. Univariate and multivariate Cox proportional hazards regression analyses were conducted to evaluate factors associated with renal outcomes. HR and 95% CI were calculated. Variables included in the multivariate model were selected based on clinical relevance and statistical significance (*p* < 0.10) in the univariate analysis. To avoid multicollinearity with PhA, HGS was excluded from the multivariate analysis.

**Table 4:** Cox proportional hazards analysis for renal outcomes using dichotomized PhA values.

Parameter	Multivariate	
	HR (95% CI)	*p* Value
Age	0.990 (0.947–1.034)	0.646
Sex (Ref. M)	0.783 (0.290–2.114)	0.630
BMI	0.981 (0.882–1.092)	0.728
sBP	0.976 (0.937–1.017)	0.253
dBP	1.005 (0.958–1.054)	0.844
eGFR	0.921 (0.884–0.959)	<0.001
Alb	0.557 (0.190–1.636)	0.287
High PhA	Ref.	–
Low PhA	4.540 (1.417–14.54)	0.011

HR, hazard ratio; CI, confidence interval; Ref, reference; M; male, BMI; body mass index, sBP; systolic blood pressure, dBP; diastolic blood pressure, eGFR; estimated glomerular filtration rate, Alb; albumin, PhA; phase angle. Cox proportional hazards regression analysis was conducted using sex-specific PhA cutoff values derived from ROC analysis (4.50° for men, 4.00° for women). PhA was entered as a dichotomous variable, with the high PhA group set as the reference. HR and 95% CI were calculated to assess the risk of renal outcomes in patients with low PhA.

## Data Availability

The data presented in this study are available on request from the corresponding author due to ethical and privacy restrictions, but are available from the corresponding author upon reasonable request.

## References

[1] Jager K.J., Kovesdy C., Langham R., Rosenberg M., Jha V., Zoccali C. (2019). A single number for advocacy and communication—Worldwide more than 850 million individuals have kidney diseases. Nephrol. Dial. Transplant..

[2] Fahal I.H. (2014). Uraemic sarcopenia: Aetiology and implications. Nephrol. Dial. Transplant..

[3] Ishikawa S., Naito S., Iimori S., Takahashi D., Zeniya M., Sato H., Nomura N., Sohara E., Okado T., Uchida S. (2018). Loop diuretics are associated with greater risk of sarcopenia in patients with non-dialysis-dependent chronic kidney disease. PLoS ONE.

[4] Mae Y., Takata T., Taniguchi S., Fujino Y., Kageyama K., Hanada H., Iyama T., Sugihara T., Isomoto H. (2025). Selective peroxisome proliferator-activated receptor-α modulator improves hypertriglyceridemia and muscle quality in patients with chronic kidney disease: A retrospective observational study. Clin. Nutr. ESPEN.

[5] Taniguchi S., Takata T., Mae Y., Fujino Y., Kageyama K., Hanada H., Iyama T., Isomoto H. (2024). Managing dosage adjustments in pseudo-hypocreatinemia: Insights from vancomycin-induced nephrotoxicity in a sarcopenic patient. Yonago Acta Med..

[6] Zheng X., Ren X., Jiang M., Han L., Zhong C. (2024). Association of Sarcopenia with Rapid Kidney Function Decline and Chronic Kidney Disease in Adults with Normal Kidney Function. Br. J. Nutr..

[7] Pereira R.A., Cordeiro A.C., Avesani C.M., Carrero J.J., Lindholm B., Amparo F.C., Amodeo C., Cuppari L., Kamimura M.A. (2015). Sarcopenia in Chronic Kidney Disease on Conservative Therapy: Prevalence and Association with Mortality. Nephrol. Dial. Transplant..

[8] Mori K., Nishide K., Okuno S., Shoji T., Emoto M., Tsuda A., Nakatani S., Imanishi Y., Ishimura E., Yamakawa T. (2019). Impact of diabetes on sarcopenia and mortality in patients undergoing hemodialysis. BMC Nephrol..

[9] Mae Y., Takata T., Yamada K., Hamada S., Yamamoto M., Iyama T., Isomoto H. (2022). Creatinine generation rate can detect sarcopenia in patients with hemodialysis. Clin. Exp. Nephrol..

[10] Takata T., Motoe A., Tanida K., Taniguchi S., Ida A., Yamada K., Hamada S., Ogawa M., Yamamoto M., Mae Y. (2021). Feasibility of computed tomography-based assessment of skeletal muscle mass in hemodialysis patients. J. Nephrol..

[11] Cruz-Jentoft A.J., Bahat G., Bauer J., Boirie Y., Bruyère O., Cederholm T., Cooper C., Landi F., Rolland Y., Sayer A.A. (2019). Sarcopenia: Revised European consensus on definition and diagnosis. Age Ageing.

[12] McGregor R.A., Cameron-Smith D., Poppitt S.D. (2014). It Is Not Just Muscle Mass: A Review of Muscle Quality, Composition and Metabolism during Ageing as Determinants of Muscle Function and Mobility in Later Life. Longev. Healthspan.

[13] Fragala M.S., Kenny A.M., Kuchel G.A. (2015). Muscle quality in aging: A multi-dimensional approach to muscle functioning with applications for treatment. Sports Med..

[14] Barbosa-Silva M.C., Barros A.J., Wang J., Heymsfield S.B., Pierson R.N. (2005). Bioelectrical impedance analysis: Population reference values for phase angle by age and sex. Am. J. Clin. Nutr..

[15] Yamada Y., Buehring B., Krueger D., Anderson R.M., Schoeller D.A., Binkley N. (2017). Electrical properties assessed by bioelectrical impedance spectroscopy as biomarkers of age-related loss of skeletal muscle quantity and quality. J. Gerontol. A Biol. Sci. Med. Sci..

[16] Akamatsu Y., Kusakabe T., Arai H., Yamamoto Y., Nakao K., Ikeue K., Ishihara Y., Tagami T., Yasoda A., Ishii K. (2022). Phase angle from bioelectrical impedance analysis is a useful indicator of muscle quality. J. Cachexia Sarcopenia Muscle.

[17] Zeng Y., Chen Y., Yang Y., Qiu Y., Fu P., Yuan H. (2024). Bioelectrical impedance analysis-derived phase angle predicts possible sarcopenia in patients on maintenance hemodialysis: A retrospective study. BMC Nephrol..

[18] Ding Y., Chang L., Zhang H., Wang S. (2022). Predictive value of phase angle in sarcopenia in patients on maintenance hemodialysis. Nutrition.

[19] Chen Y., Wu J., Ran L., Yu D., Chen X., Liu M. (2022). The combination of phase angle and age has a good diagnostic value for sarcopenia in continuous ambulatory peritoneal dialysis patients. Front. Nutr..

[20] Wang Y., Hu Y., Zhang M., Jin H., Wen Y., Tang R., Wang B., Liu B., Liu H. (2023). Bioelectrical impedance analysis-derived phase angle predicts sarcopenia in patients on maintenance hemodialysis. Nutr. Clin. Pract..

[21] Bae E., Lee T.W., Bae W., Kim S., Choi J., Jang H.N., Chang S.H., Park D.J. (2022). Impact of phase angle and sarcopenia estimated by bioimpedance analysis on clinical prognosis in patients undergoing hemodialysis: A retrospective study. Medicine.

[22] Matsuo S., Imai E., Horio M., Yasuda Y., Tomita K., Nitta K., Yamagata K., Tomino Y., Yokoyama H., Hishida A. (2009). Revised equations for estimated GFR from serum creatinine in Japan. Am. J. Kidney Dis..

[23] Coresh J., Turin T.C., Matsushita K., Sang Y., Ballew S.H., Appel L.J., Arima H., Chadban S.J., Cirillo M., Djurdjev O. (2014). Decline in estimated glomerular filtration rate and subsequent risk of end-stage renal disease and mortality. JAMA.

[24] Matsushita K., Chen J., Sang Y., Ballew S.H., Shimazaki R., Fukagawa M., Imai E., Coresh J., Hishida A. (2016). Risk of end-stage renal disease in Japanese patients with chronic kidney disease increases proportionately to decline in estimated glomerular filtration rate. Kidney Int..

[25] Chen L.K., Woo J., Assantachai P., Auyeung T.W., Chou M.Y., Iijima K., Jang H.C., Kang L., Kim M., Kim S. (2020). Asian Working Group for Sarcopenia: 2019 Consensus Update on Sarcopenia Diagnosis and Treatment. J. Am. Med. Dir. Assoc..

[26] Gonzalez M.C., Barbosa-Silva T.G., Bielemann R.M., Gallagher D., Heymsfield S.B. (2016). Phase angle and its determinants in healthy subjects: Influence of body composition. Am. J. Clin. Nutr..

[27] Norman K., Stobäus N., Pirlich M., Bosy-Westphal A. (2012). Bioelectrical phase angle and impedance vector analysis—Clinical relevance and applicability of impedance parameters. Clin. Nutr..

[28] Beberashvili I., Azar A., Sinuani I., Shapiro G., Feldman L., Stav K., Sandbank J., Averbukh Z. (2014). Bioimpedance phase angle predicts muscle function, quality of life and clinical outcome in maintenance hemodialysis patients. Eur. J. Clin. Nutr..

[29] Basile C., Della-Morte D., Cacciatore F., Gargiulo G., Galizia G., Roselli M., Curcio F., Bonaduce D., Abete P. (2014). Phase angle as bioelectrical marker to identify elderly patients at risk of sarcopenia. Exp. Gerontol..

[30] Kyle U.G., Soundar E.P., Genton L., Pichard C. (2012). Can phase angle determined by bioelectrical impedance analysis assess nutritional risk? A comparison between healthy and hospitalized subjects. Clin. Nutr..

[31] Lukaski H.C., Kyle U.G., Kondrup J. (2017). Assessment of adult malnutrition and prognosis with bioelectrical impedance analysis: Phase angle and impedance ratio. Curr. Opin. Clin. Nutr. Metab. Care.

[32] Kosacka M., Korzeniewska A., Jankowska R. (2013). The evaluation of body composition, adiponectin, C-reactive protein and cholesterol levels in patients with obstructive sleep apnea syndrome. Adv. Clin. Exp. Med..

[33] Moon S.J., Kim T.H., Yoon S.Y., Chung J.H., Hwang H.J. (2015). Relationship between Stage of Chronic Kidney Disease and Sarcopenia in Korean Aged 40 Years and Older Using the Korea National Health and Nutrition Examination Surveys (KNHANES IV-2, 3, and V-1, 2), 2008–2011. PLoS ONE.

[34] Ren H., Gong D., Jia F., Xu B., Liu Z. (2016). Sarcopenia in patients undergoing maintenance hemodialysis: Incidence rate, risk factors and its effect on survival risk. Ren. Fail..

[35] Kim J.K., Choi S.R., Choi M.J., Kim S.G., Lee Y.K., Noh J.W., Kim H.J., Song Y.R. (2014). Prevalence of and factors associated with sarcopenia in elderly patients with end-stage renal disease. Clin. Nutr..

[36] Hortegal E.V.F., Alves J.J.D.A., Santos E.J.F., Nunes L.C.R., da Silva J.C.G., Nunes R.F., de Almeida Melo D., de Carvalho S.C.R., da Cunha França A.K.T., dos Santos E.M. (2020). Sarcopenia and inflammation in patients undergoing hemodialysis. Nutr. Hosp..

